# Case Report: Occult gastric corrosion of a brass pendant during endoscopic retrieval in a child

**DOI:** 10.3389/fped.2026.1765313

**Published:** 2026-02-17

**Authors:** Genki Ehara, Miri Nomura, Yukari Mizoguchi, Ryo Kimura, Daisuke Yamaguchi, Toshihiko Kakiuchi

**Affiliations:** 1 Department of Pediatrics, Faculty of Medicine, Saga University, Saga, Japan; 2 Division of Gastroenterology, Department of Internal Medicine, Faculty of Medicine, Saga University, Saga, Japan

**Keywords:** child, corrosion, endoscopy, esophageal diseases, foreign bodies, gastrointestinal, preschool

## Abstract

**Background:**

Ingestion of a metallic foreign body is common among young children. Although most objects pass spontaneously, some require endoscopic removal. Brass and similar metals can undergo acid-induced dezincification, leading to internal weakening even if the surface appears unchanged. This report describes a rare case of brass pendant ingestion affected by such corrosion.

**Case presentation:**

A 4-year-old boy ingested a brass pendant that remained in his stomach for 72 h, prompting endoscopic retrieval. Although there was no visible corrosion, the pendant fractured at a narrow segment when traction was applied at a physiological esophageal narrowing site. The remaining portion was safely removed after re-grasping the thickest, structurally strongest region.

**Conclusion:**

Despite having a normal appearance, metallic foreign bodies may weaken internally after 48–72 h (2–3 days) of gastric exposure. Hence, endoscopists should anticipate hidden corrosion and grasp the thickest, most reinforced area during removal while considering mechanical stress at esophageal narrowing sites to ensure safe retrieval.

## 1 Introduction

Foreign body ingestion is a common cause of pediatric emergency evaluation among pre-school age children (1, 2), who characteristically exhibit oral exploratory behavior as part of normal neurodevelopment. This developmental tendency predisposes young children to place small objects in their mouths, thereby increasing the risk of accidental ingestion. Metallic objects generally pass spontaneously. However, endoscopic retrieval is required in 10%–20% of cases (3). Current guidelines recommend the removal of sharp or hazardous objects, as well as objects wider than 2.5 cm in diameter or longer than 6 cm, depending on their characteristics (3–5). Certain metallic foreign bodies require prompt endoscopic retrieval because of their potential to cause rapid tissue injury or chemical degradation. Button batteries are particularly hazardous, as they can generate electrical currents and release alkaline substances, leading to severe mucosal injury (6). In addition, objects composed of zinc- or nickel-containing alloys are susceptible to gastric acid–induced corrosion and metal ion release, with the potential for toxicity, thereby warranting early endoscopic intervention (7).

When exposed to gastric acid, metals such as brass, copper alloys, and zinc-containing materials are susceptible to corrosion. One important mechanism is *dezincification*, a selective leaching of zinc that leaves behind a porous and mechanically fragile copper-rich matrix. The early stages of this process may occur without evident surface color change, pitting, or texture irregularity. Toxicology and material science literature has described corrosion of ingested objects. However, only a few clinical reports have shown its impact on endoscopic removal in children (8). Current pediatric endoscopy guidelines do not address corrosion-induced internal weakening or its implications for endoscopic retrieval strategies (4).

Herein, we present a case in which a brass pendant, visually normal after 3 days of gastric retention, fractured during removal due to occult weakening. This case shows the importance of anticipating structural degradation and selecting appropriate grasping points during endoscopic retrieval of retained metallic objects.

## 2 Case description

**Figure 1:**
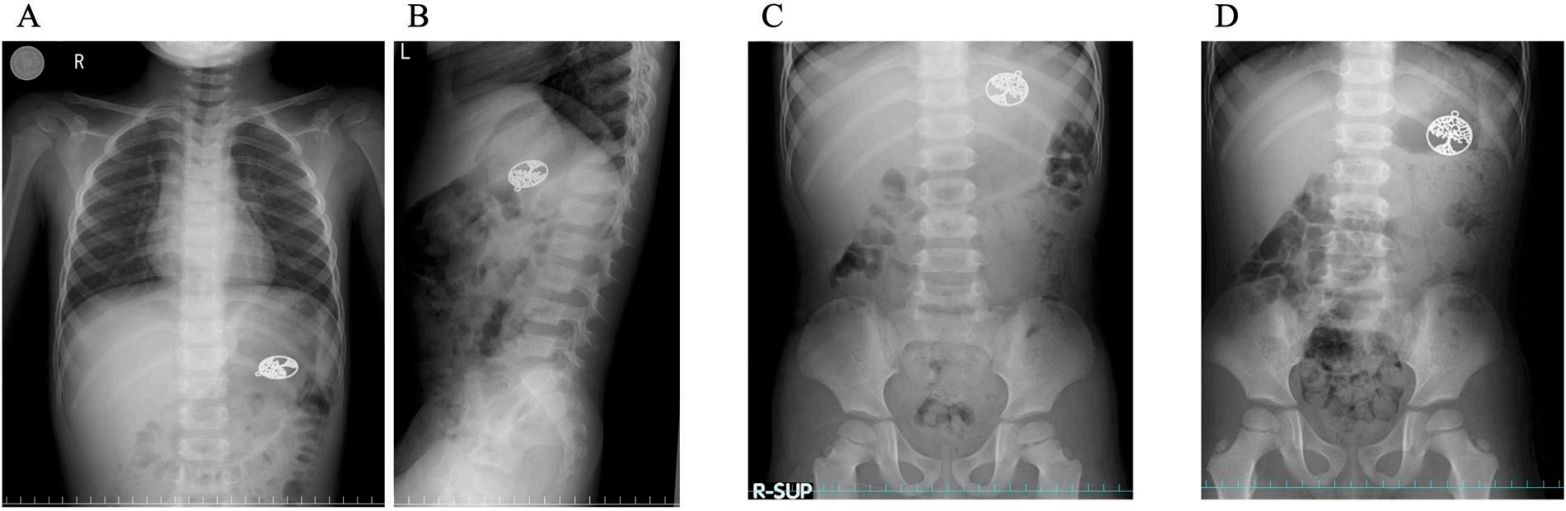
The time course of a swallowed metal object in a patient's stomach. The object was located in the stomach immediately after ingestion **(A,B)**, at 24 h **(C)**, and at 72 h **(D)** however, it did not pass into the small intestine.

A healthy 4-year-old boy accidentally ingested a brass pendant from a necklace. The pendant was roughly circular, measuring approximately 3 cm in diameter and 2 mm in thickness. The assessment of the pendant's composition was based on its external appearance and the manufacturer's description, and was not confirmed by chemical analysis. At the time of ingestion, the object was visually intact, with no signs of corrosion. Radiography performed at our institution confirmed the presence of a metallic object in the stomach (Figures 1A,B). As the child remained asymptomatic, he was monitored for 72 h. Serial radiography revealed no progression beyond the stomach (Figures 1C,D), prompting endoscopic removal after guideline-based recommendations (3–5).

**Figure 2:**
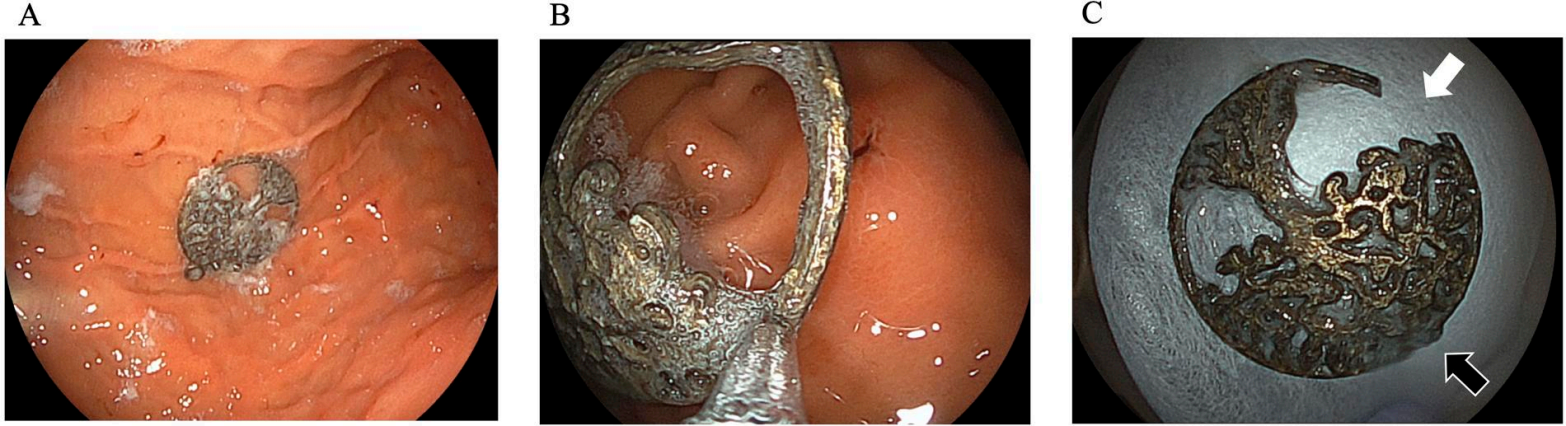
Endoscopic findings showed that the foreign body was located in the stomach body **(A)** during the initial removal, it was grasped at the easiest point **(B)** eventually, it was grasped at a different point and was successfully removed. The part of the metal foreign body that was initially grasped had broken **(C)** white arrow, first grasping position; Black arrow, second grasping position.

The pendant was located in the gastric body during upper endoscopy (Figure 2A). Despite 3 days of gastric exposure, the pendant's appearance remained almost unchanged. There was no visible discoloration, pitting, or textural alteration. The endoscopist selected the narrowest part of the pendant for grasping, anticipating easier passage through the esophagus (Figure 2B). Upper gastrointestinal endoscopy was performed using a standard pediatric diagnostic gastroscope (Olympus, Tokyo, Japan) with an external diameter of approximately 5.9–6.0 mm and a 2.0-mm working channel, which is routinely used for foreign body retrieval in preschool-aged children. The foreign body was initially grasped using standard grasping forceps compatible with the 2.0-mm working channel.

During withdrawal, resistance was detected at one of the esophagus's physiological narrowing sites. At this point, the grasped segment abruptly fractured, consistent with a compromised structural integrity possibly caused by gastric acid-induced dezincification. The fragment was successfully retrieved, and the remaining portion fell back into the stomach.

The endoscopist re-evaluated the pendant's morphology, paying special attention to its thickness and structural reinforcement. During the second attempt, the thickest central portion was grasped using a grasping forceps, distributing traction forces more evenly. Using this approach, a successful extraction was achieved without further fragmentation or mucosal injury (Figure 2C). The pendant fractured during the first extraction attempt (Figure 2C, white arrow), whereas no apparent damage was observed at the site grasped during the second, successful extraction (Figure 2C, black arrow). The gross appearance of the retrieved pendant was consistent with the endoscopic findings, and no obvious discoloration or surface deterioration was noted (Figure 2C). The patient recovered uneventfully, and he was discharged the next day.

## 3 Discussion

This case emphasizes a clinically important but under-recognized phenomenon: metallic objects that appear intact during endoscopy may, nonetheless, be structurally compromised after prolonged gastric exposure. In particular, brass, a copper–zinc alloy, is susceptible to acid-induced corrosion. *Dezincification* occurs if zinc dissolves out of the alloy in acidic environments, producing a weakened, porous copper-rich layer (8). This process can occur within 48–72 h under conditions that simulate gastric acid exposure (7). Notably, this degradation may progress internally before the appearance of visible surface changes, such as discoloration and pitting. In our case, the pendant's surface appeared almost intact. However, the initially grasped narrow segment fractured during extraction, strongly indicating internal weakening.

Mechanical factors also play an important role in the complication observed. The esophagus has natural anatomic narrowing sites—such as that at the upper esophageal sphincter and the aortic arch level—where resistance increases during retrieval (9, 10). During extraction, resistance is commonly encountered at physiological esophageal narrowing sites, including the upper esophageal sphincter and the level of the aortic arch. Passage through these regions increases mechanical resistance and concentrates traction forces, which may exceed the tensile strength of a structurally compromised metallic object, thereby predisposing it to fragmentation. Traction applied to a structurally weakened segment may exceed its compromised tensile strength when passing through these points. The initial decision to grasp the pendant at its narrowest point, although logical under typical circumstances, concentrated mechanical forces on the area most likely to have undergone acid-induced weakening.

This incident emphasizes the importance of grasping strategy during endoscopic retrieval. The guidelines emphasize the cautious selection of retrieval devices and safe removal techniques (3–5). However, they do not address structural changes that metallic objects may undergo during gastric retention (11). Our case shows that if metallic objects remain in the stomach for several days, it is advisable to grasp the thickest or most structurally reinforced portion of the object, even if this site is less convenient for extraction. A robust grasp reduces the risk of fragmentation, decreases mucosal trauma, shortens procedure time, and prevents the need for repeat sedation. At our institution, the selection of retrieval devices is individualized based on the shape, size, and presumed structural integrity of the foreign body. Atraumatic adjuncts, such as retrieval nets or protective strategies, are selectively employed when appropriate. However, when structural weakening is suspected—particularly in zinc-containing alloys—these devices may increase stress concentration and the risk of fragmentation. In the present case, grasping forceps allowed controlled reassessment of the object and adjustment of the grasping point after fracture occurred.

In addition, early retrieval of metallic foreign bodies may reduce corrosion-related risks. Although monitoring is appropriate in several cases, metals such as brass and copper alloys are vulnerable to gastric conditions (7). If removal is delayed, clinicians should anticipate the risk of hidden corrosion and adjust their retrieval strategy accordingly. If fragmentation occurs, as in this case, reassessment of the object's structural features and selection of a stronger grasping point are essential. In this case, the patient was asymptomatic, and the foreign body was not sharp but was over 2.5 cm in diameter, so the foreign body was removed endoscopically after 72 h of intragastric retention without waiting 4 weeks, as per current pediatric guidelines. Experimental and *in vitro* studies suggest that acid-induced dezincification of zinc-containing alloys can occur within 48–72 h (7), and the clinically significant weakening observed in this case may therefore have developed within the documented 72-hour period, suggesting that earlier removal may be warranted in similar cases.

This case contributes to clinical understanding by showing that corrosion-related weakening can occur rapidly, remain visually undetectable, and directly influence the success and safety of endoscopic retrieval. Importantly, visual inspection alone is insufficient to exclude internal corrosion in zinc-containing alloys. Even in the absence of surface discoloration, pitting, or textural changes, substantial internal degradation may be present and directly affect the safety of endoscopic retrieval. Awareness of this limitation is essential when planning the grasping strategy and extraction technique. Greater awareness of these issues may help prevent complications in similar pediatric cases.

In conclusion, metallic foreign bodies retained in the stomach for several days may undergo acid-induced corrosion that weakens their structural integrity without causing visible changes. Hence, endoscopists should be aware of this possibility and consider grasping thicker, more robust portions of the object during retrieval. Anticipating mechanical stress at esophageal narrowing sites and adapting techniques accordingly can help ensure a safe and effective removal.

## Data Availability

The original contributions presented in the study are included in the article/Supplementary Material, further inquiries can be directed to the corresponding author.
